# Transverse Colon Injury During Initial Umbilical Access for Robot-Assisted Laparoscopic Hysterectomy: Presumed Bowel Tethering by Omental Adhesions to the Abdominal Wall

**DOI:** 10.7759/cureus.116682

**Published:** 2026-09-21

**Authors:** Tadahisa Takeuchi, Noriko Ikeda, Kouta Nakano, Hiroyasu Nakajima, Hachidai Hirakawa

**Affiliations:** 1 Department of Obstetrics and Gynecology, Oita Prefectural Hospital, Oita, JPN

**Keywords:** bowel injury, laparoscopic entry, omental adhesion, robot-assisted surgery, transverse colon, trocar injury, visceral slide

## Abstract

Initial laparoscopic access can cause visceral injury even in patients without previous abdominal surgery. Here, we report the case of a 49-year-old woman with adenomyosis and no history of abdominal surgery underwent robot-assisted laparoscopic hysterectomy with bilateral salpingectomy. During initial umbilical access, a camera port fitted with a reusable 8-mm blunt obturator was advanced through the peritoneum. Immediately after camera insertion, bowel mucosa was visualized before pneumoperitoneum had been established. Mini-laparotomy revealed omental adhesion to the anterior abdominal wall, whereas the transverse colon itself was not directly adherent. Two closely adjacent perforations of the transverse colon were consistent with tangential penetration of the colonic wall after the instrument had passed through the adherent omentum. The injury was repaired in two layers, and the planned robotic procedure was completed by converting the mini-laparotomy incision into a sealed camera-port site. Recovery was uneventful. The adherent omentum was considered to have tethered the transverse colon close to the abdominal wall. This case illustrates that the absence of previous abdominal surgery does not guarantee safe umbilical access. Controlling the force and depth of peritoneal entry, using preoperative ultrasonographic assessment as an adjunct rather than a guarantee of safety, and immediately recognizing and systematically evaluating any access-related bowel injury may help reduce the risk of injury and limit its consequences.

## Introduction

Initial abdominal access is a brief but potentially hazardous step in laparoscopic and robot-assisted gynecologic surgery. Although bowel injury is uncommon, delayed recognition substantially worsens outcomes; a systematic review of bowel injuries during gynecologic laparoscopy found that all reported deaths occurred after delayed diagnosis [1]. Previous abdominal surgery is an important predictor of anterior abdominal wall adhesions, and alternative entry sites are commonly considered in patients with high-risk surgical histories. However, clinically relevant umbilical adhesions can also occur in patients without previous abdominal surgery [2].

Appropriate port positioning is important in robot-assisted hysterectomy because it affects instrument access and the risk of robotic-arm collision [3]. An access-related complication at the initial camera-port site may therefore disrupt the planned robotic setup. We report a transverse colon injury that occurred during initial umbilical access for robot-assisted laparoscopic hysterectomy in a patient with no previous abdominal surgery. The transverse colon was not directly adherent to the abdominal wall; instead, the operative findings suggested that omental adhesion to the abdominal wall tethered the colon near the umbilicus, allowing the access instrument to pass through the omentum and penetrate the colonic wall. We present this case to highlight that indirect bowel tethering by adherent omentum may place bowel near an umbilical access site even in a patient without previous abdominal surgery, and to describe the presumed mechanism of injury, intraoperative recovery strategy, and subsequent revision of our initial access technique.

## Case presentation

A 49-year-old Asian woman of Japanese nationality, gravida 6 para 5 (five vaginal deliveries and one induced abortion), presented with dysmenorrhea and was diagnosed with adenomyosis. She had no history of abdominal surgery and no history of appendicitis, pelvic peritonitis, endometriosis, or other conditions that could predispose to intra-abdominal adhesions. Her height was 163 cm, weight 77 kg, and body mass index was 29.0 kg/m². Conservative treatment did not adequately relieve her symptoms, and she elected to undergo hysterectomy.

After the risks of perioperative complications had been explained and informed consent had been obtained, robot-assisted laparoscopic hysterectomy with bilateral salpingectomy using the da Vinci Xi Surgical System (Intuitive Surgical, Inc., Sunnyvale, California, United States) was planned.

Under general anesthesia, the patient was placed in a legs-apart position, and intermittent pneumatic compression devices were applied to both lower legs. After confirming that she tolerated a 20° Trendelenburg position, a uterine manipulator and an indwelling bladder catheter were inserted. A 20-mm skin incision was made at the umbilicus. A Veress needle was not used. The fascial defect at the umbilical ring was identified under direct vision, and the fascia was grasped and elevated. The planned technique was to advance the camera port fitted with a reusable 8-mm blunt obturator (470008; Intuitive Surgical, Inc.), bluntly penetrate the peritoneum, and enter the abdominal cavity. Pneumoperitoneum was to be initiated only after insertion of the camera and confirmation of intraperitoneal placement. 

When the camera was inserted, intestinal mucosa was seen in the field of view (Figure 1). The robot had not yet been docked, and pneumoperitoneum had not been established. Bowel injury was immediately suspected, and a gastrointestinal surgeon was immediately called to assist with repair.

**Figure 1:**
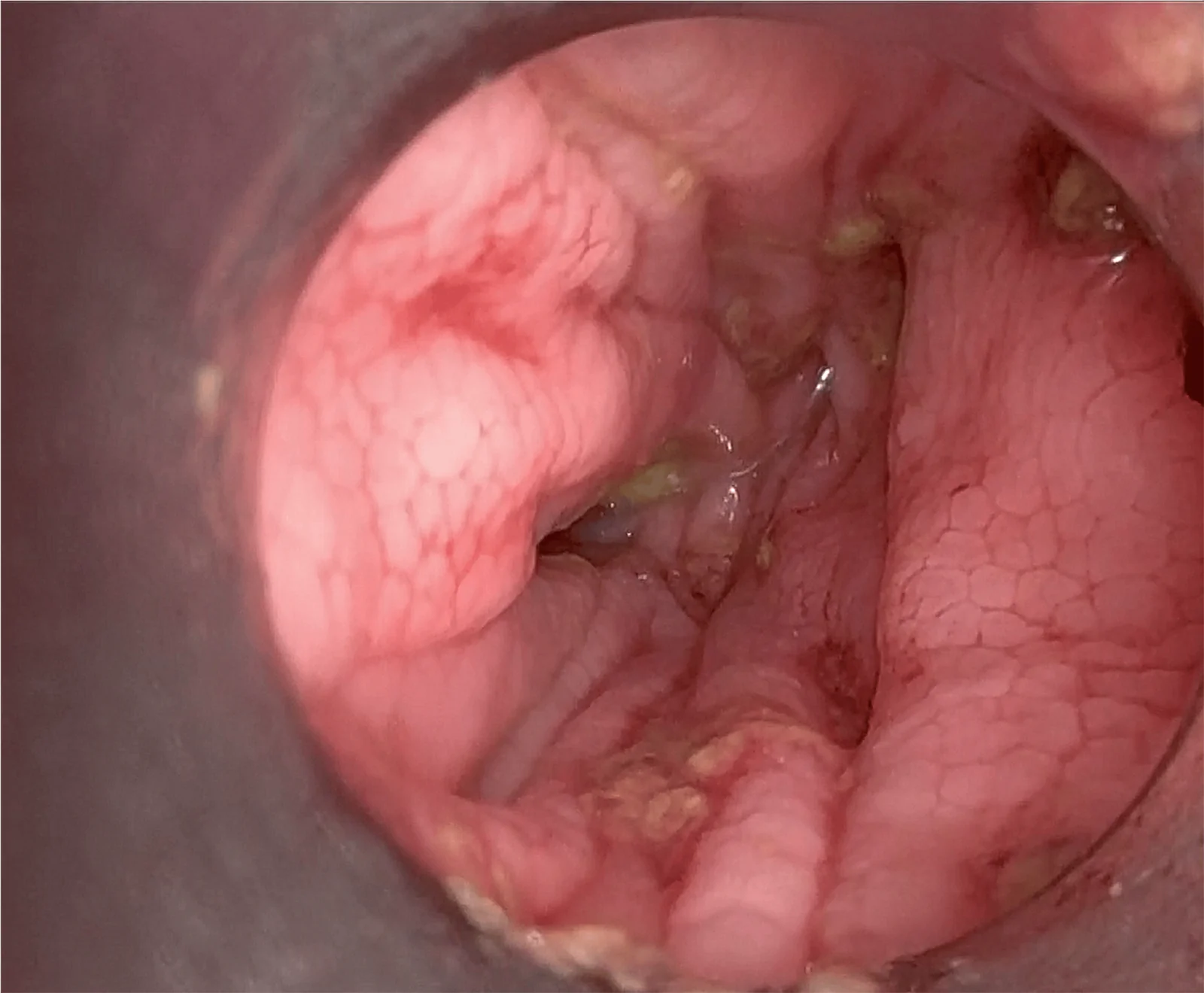
Endoscopic view after camera insertion Endoscopic view immediately after camera insertion showing intestinal mucosa before pneumoperitoneum was established.

The umbilical skin incision was extended craniocaudally to 5 cm, and the fascia and peritoneum were identified cranially to gain access to the abdominal cavity. Omental adhesions to the anterior abdominal wall were present at the umbilicus and were dissected only to the extent necessary for repair. The transverse colon itself was not directly adherent to the anterior abdominal wall at the umbilicus.

A Lap-Protector (Hakko Co., Ltd., Chikuma-shi, Nagano, Japan) was placed in the mini-laparotomy incision for inspection of the abdominal cavity. The omentum was reflected cranially, and the entire small bowel was examined from the ligament of Treitz to the terminal ileum; no obvious injury was identified. Examination of the transverse colon then revealed two closely adjacent perforations near the omental attachment in the mid-transverse colon, consistent with tangential penetration of the colonic wall. The trocar was considered to have passed through the adherent omentum before penetrating the transverse colon. The tissue bridge between the two perforations was divided to create a single defect, with a maximum diameter of approximately 1.5 cm (Figure 2).

**Figure 2:**
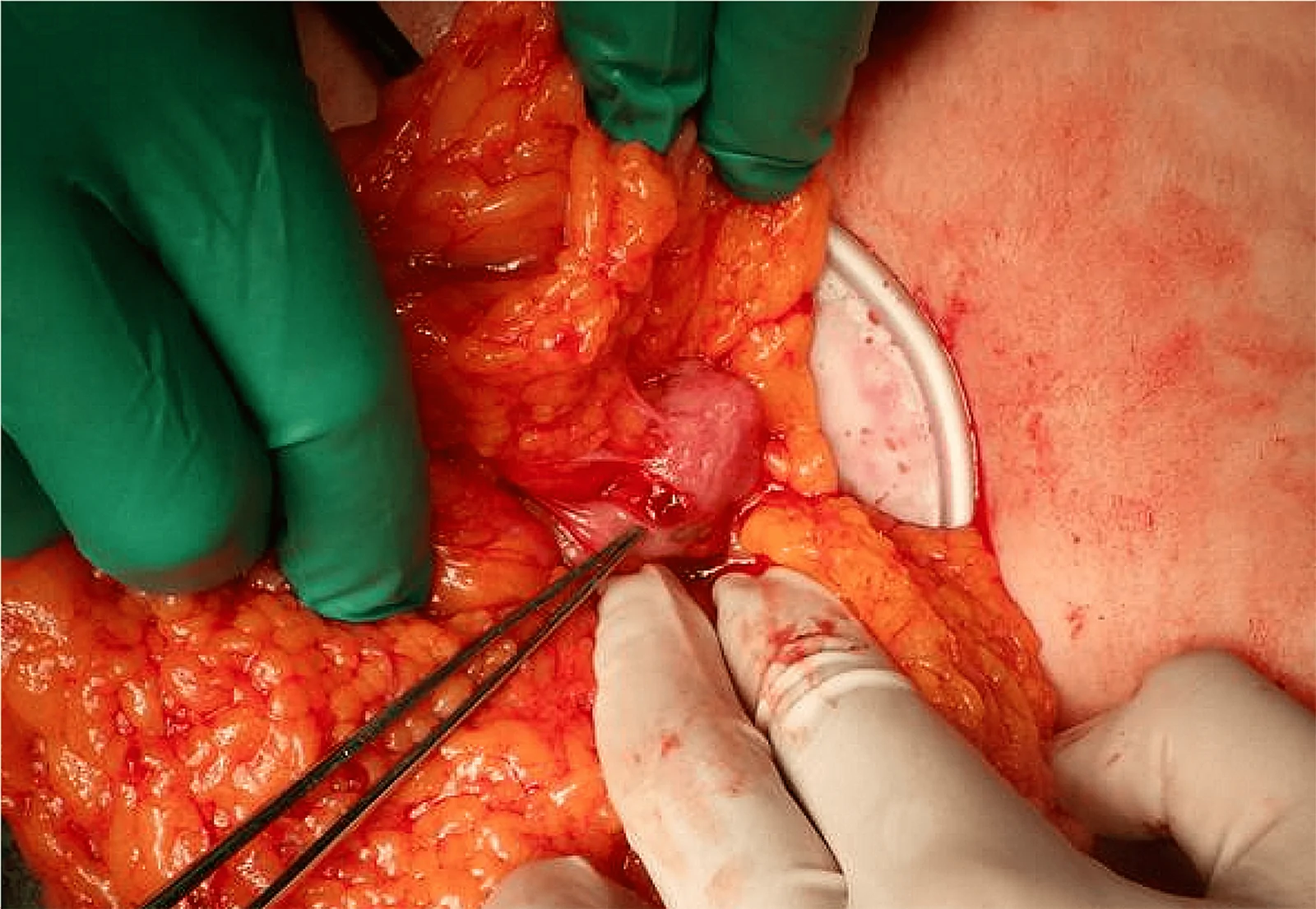
Transverse colon injury Transverse colon injury near the omental attachment after division of the tissue bridge between two closely adjacent perforations. The tissue bridge was divided to create a single defect for repair. The forceps tip indicates the defect. The original two perforations were considered consistent with tangential penetration of the colonic wall.

The injury was closed in two layers using 4-0 polydioxanone (PDS), with a full-thickness layer followed by a seromuscular layer. Neither bowel resection nor stoma formation was required. Contamination was limited and minimal, and the area around the repair was irrigated with normal saline.

Because the injury was localized, contamination was minimal, and repair had been completed by the surgeon with a two-layer closure, continuation of the planned procedure was considered feasible. An EZ Access device (Hakko Co., Ltd.) was attached to the Lap-Protector, and an 8-mm da Vinci trocar was inserted through this site to establish pneumoperitoneum (Figure 3).

**Figure 3:**
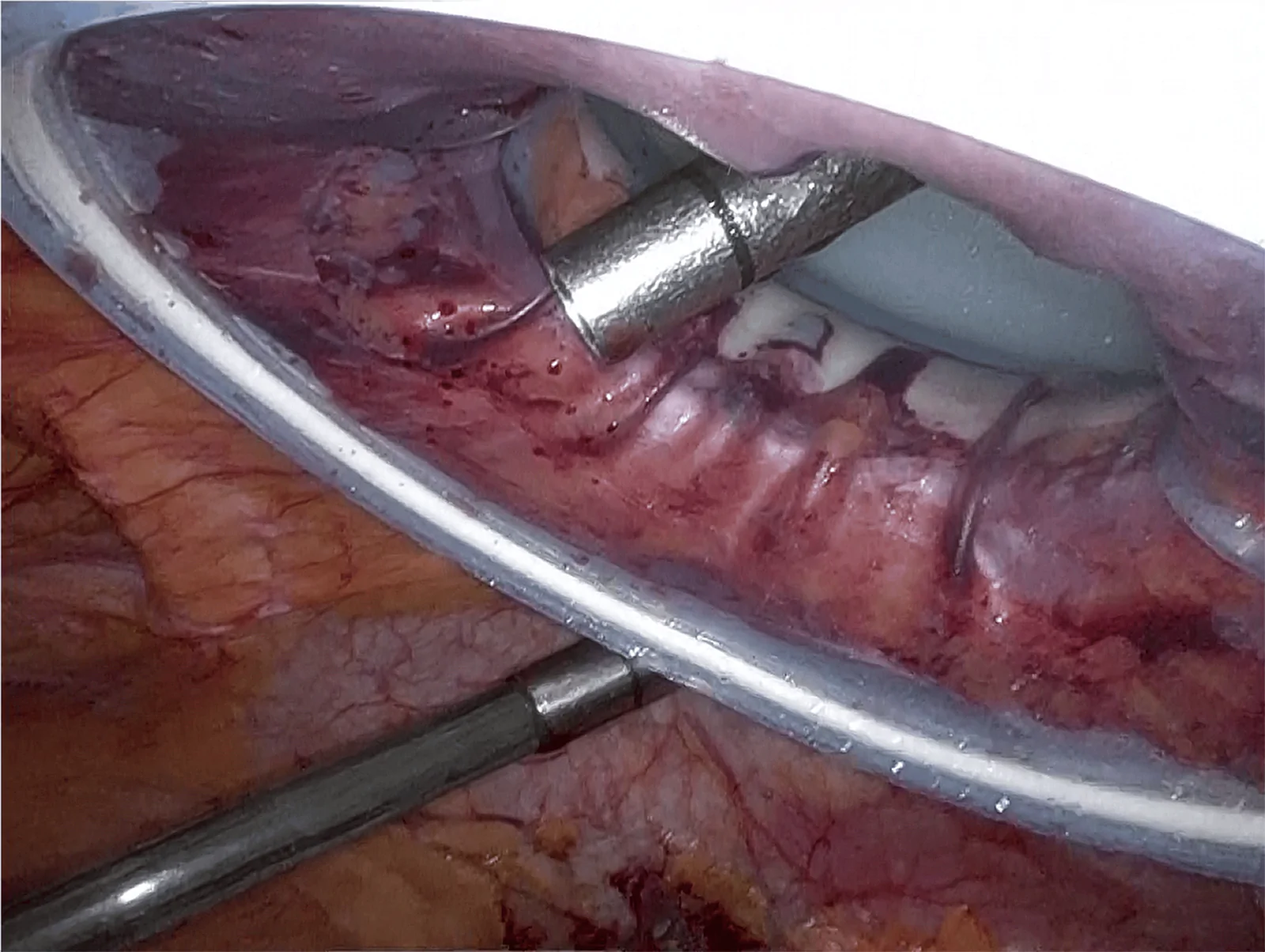
EZ Access mounted on the mini-laparotomy incision Lap-Protector and EZ Access (Hakko Co., Ltd., Nagano, Japan) mounted on the mini-laparotomy incision, allowing insertion of an 8-mm da Vinci trocar (Intuitive Surgical, Inc., Sunnyvale, California, United States) and establishment of pneumoperitoneum.

All subsequent ports were placed under laparoscopic visualization. The port configuration was redesigned using the mini-laparotomy incision as the camera port. A da Vinci port was placed to the left of the umbilicus under laparoscopic visualization, and residual adhesions between the peritoneum and omentum in the right upper abdomen were released with a laparoscopic monopolar electrosurgical instrument introduced through this port. The remaining required da Vinci ports and a 12-mm assistant port were then placed under laparoscopic visualization. After port placement, intra-abdominal inspection confirmed that the omentum was adherent to the anterior abdominal wall from the umbilicus to the right upper abdomen (Figure 4).

**Figure 4:**
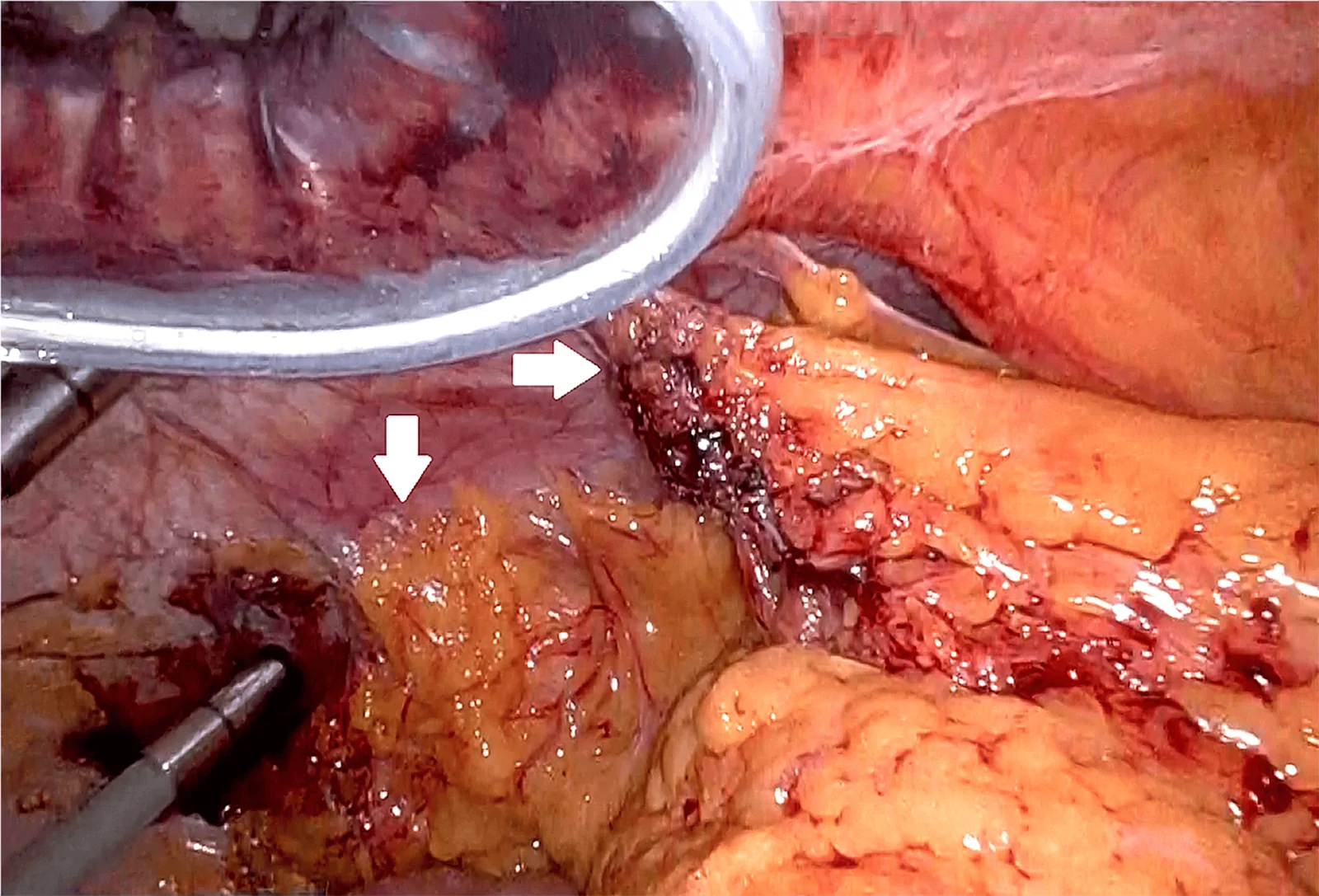
Omental adhesion (arrows) to the anterior abdominal wall Omental adhesion to the anterior abdominal wall extending from the umbilicus toward the right upper abdomen.

After docking, adhesions between the omentum and right pelvic peritoneum were released to allow the bowel to be displaced cranially, and robot-assisted hysterectomy with bilateral salpingectomy was then completed in the usual manner. The uterus and both fallopian tubes were removed vaginally, and a 15-Fr drain was placed in the pelvis. The fascia at the umbilical incision and the 12-mm assistant-port site was closed. Total operative time was 4 hours 49 minutes, including 1 hour 55 minutes before docking, and estimated blood loss was approximately 60 mL. The excised uterus weighed 259 g.

Perioperative cefmetazole was administered at 1 g twice daily through postoperative day 3. Oral intake was resumed on postoperative day 5, and the pelvic drain was removed on postoperative day 7. No postoperative complications, including leakage from the bowel repair, intra-abdominal abscess, or surgical-site infection, occurred, and the patient was discharged on postoperative day 10.

## Discussion

The frequency of umbilical adhesions varies markedly not only according to the presence or absence of previous abdominal surgery but also according to the type of prior surgery. Audebert and Gomel directly inspected the umbilicus by microlaparoscopy from the left upper quadrant in 814 patients divided into four groups according to surgical history [2]. They reported umbilical adhesions involving the omentum or bowel in 0.68% of 469 patients with no previous abdominal surgery, 1.6% of 125 patients with previous laparoscopy only, 19.8% of 131 patients with previous laparotomy through a suprapubic transverse incision, and 51.7% of 89 patients with previous midline laparotomy. Similarly, in a prospective study of 215 patients, Levrant et al. found anterior abdominal wall adhesions in 17 of 29 patients (59%) with a previous midline vertical incision and in 11 of 39 patients (28%) with a previous suprapubic transverse incision, whereas no adhesions were found in 91 patients with no previous abdominal surgery or in 45 patients with previous laparoscopy only [4]. Thus, a history such as a previous midline incision can prompt consideration of a nonumbilical entry site.

Childers et al. used left upper quadrant entry in 41 patients at high risk for infraumbilical adhesions and identified adhesions in 28 patients (68%) [5]. However, in the study by Audebert and Gomel, severe adhesions considered to pose a potential risk of bowel injury during blind umbilical trocar insertion were identified in 40 of 814 patients (4.91%) overall and in 0.42% of patients with no previous abdominal surgery [2]. Stratification based on surgical history may prompt use of an alternative entry site, but such a trigger is less likely to exist in patients without previous abdominal surgery. Our patient also had no history of abdominal surgery, yet the omentum was adherent to the anterior abdominal wall from the umbilicus to the right upper abdomen and was additionally adherent to the right pelvic peritoneum. Nonoperative inflammatory or other intra-abdominal processes may also result in adhesions, but no clear cause was identified in this patient.

Techniques for initial laparoscopic access include the closed technique using a Veress needle, direct trocar insertion, the Hasson open technique, optical entry, and nonumbilical approaches [6]. Each has advantages and disadvantages, and because major vascular and visceral injuries are rare, no single technique has been consistently established as the safest [6,7]. In a systematic review and meta-analysis of 25 randomized controlled trials involving 6,950 patients, Raimondo et al. found lower odds of visceral injury (OR 0.17) and trocar-site infection (OR 0.27) with direct trocar insertion than with the open technique and supported direct trocar insertion as an option [8]. In contrast, a 2019 Cochrane review analyzing 57 randomized controlled trials involving 9,865 patients concluded that the evidence was insufficient to establish the superiority of any single access technique for major vascular or visceral injury; in addition, many trials excluded patients with a history of abdominal surgery or a high body mass index [7]. Therefore, in the rare anatomical setting of unexpected umbilical adhesions, no particular access technique can be assumed to reliably prevent injury.

The organs involved in adhesions to the anterior abdominal wall are relevant to the type of tissue that an instrument may first encounter during initial access. In the study by Levrant et al., 96% of anterior abdominal wall adhesions involved the omentum and 29% involved bowel [4]. This distribution, however, was derived mainly from patients with previous laparotomy, and whether the same proportions apply to patients without previous abdominal surgery is uncertain. These findings show that omental and bowel involvement in anterior abdominal wall adhesions can coexist. In our case, the operative findings suggested a related but distinct configuration: the transverse colon itself was not directly adherent to the abdominal wall; rather, it was thought to have been tethered near the umbilicus by omentum adherent to the abdominal wall, allowing the instrument to pass through the omentum and subsequently reach the colon.

With the technique used in our department at that time, the fascial defect at the umbilical ring was approached under direct vision, but the port fitted with an 8-mm blunt obturator itself was used to cross the peritoneum, making peritoneal penetration blind. The blunt obturator had a rounded, dome-shaped tip and differed in tip geometry from the single-use 8-mm bladeless obturator (optical) (470359; Intuitive Surgical, Inc.) that was introduced later (Figure 5).

**Figure 5:**
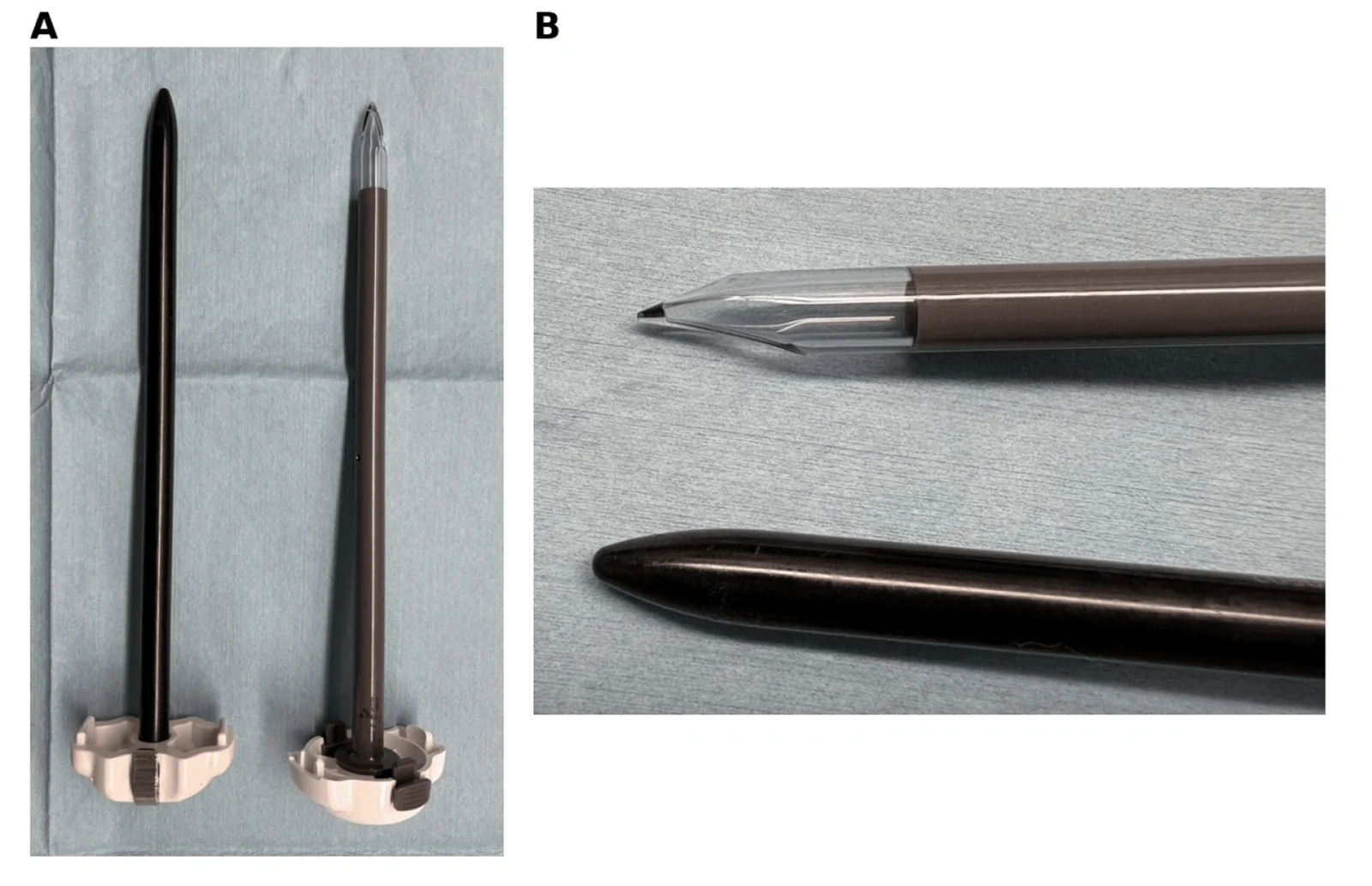
Comparison of blunt and bladeless obturators (A) Overall view of the reusable 8-mm blunt obturator (470008; Intuitive Surgical, Inc., Sunnyvale, California, United States) used at the time of injury (left) and the subsequently adopted single-use 8-mm bladeless obturator (optical) (470359; Intuitive Surgical, Inc.) (right). (B) Close-up of the tips, with the bladeless obturator above and the blunt obturator below, demonstrating their different tip geometries. Image credit: Tadahisa Takeuchi

In a porcine model, Böhm et al. compared 19 trocar designs and demonstrated that insertion force varied according to tip geometry, that conical tips required greater insertion force than pyramidal tips, and that the size of the abdominal wall defect was inversely correlated with insertion force [9]. However, the tip designs evaluated in that study were not identical to the two obturators used in our case, and actual insertion force was not measured in this patient. Therefore, a causal relationship between instrument geometry and the injury cannot be established. Nevertheless, the axial force required to penetrate the abdominal wall may have contributed to excessive advancement when tissue resistance was suddenly lost.

Preoperative assessment of abdominal wall adhesions using transabdominal ultrasonographic observation of visceral slide has been reported. In a systematic review by Limperg et al., among 12 studies involving 890 patients that evaluated periumbilical bowel adhesions, ultrasonography had a sensitivity of 95.9%, specificity of 93.1%, positive predictive value of 60.4%, and negative predictive value of 99.2% [10]. The high negative predictive value suggests that this technique may be more useful as an adjunct for identifying areas with a low probability of bowel adhesions than as a test for confirming their presence. However, visceral slide primarily assesses relative movement between the abdominal wall and underlying viscera and does not reliably establish which structure is adherent. Moreover, although the negative predictive value is high, it is not 100%, and diagnostic performance in low-risk patients without previous abdominal surgery has not been sufficiently established. No such preoperative assessment was performed in our patient. Given the adhesion of the omentum to the umbilical peritoneum and the presumed tethering of the transverse colon, visceral slide may have been reduced, but this remains a retrospective inference. Thus, preserved visceral slide on ultrasonography does not completely exclude adhesions.

Conversely, when the slide is absent or equivocal, rather than estimating which organ is adherent and proceeding blindly, we consider direct visual opening of the peritoneum more appropriate. In this case, because the initial port site became a 5-cm mini-laparotomy incision, the originally planned port arrangement could not be used unchanged, and the robotic port configuration had to be redesigned.

In robot-assisted surgery, the relative positions of the ports and the docking direction affect instrument operation; therefore, a complication during initial access directly influences subsequent setup. By mounting a Lap-Protector and EZ Access on the incision used for bowel repair, we were able to repurpose the incision as a camera-port site while maintaining pneumoperitoneum and completing the planned operation. Although these devices were not designed specifically for recovery after an access-related complication, they provided a practical means of converting the mini-laparotomy incision into a port site.

In this case, the decision to continue the planned procedure was individualized according to the extent of injury, degree of intraperitoneal contamination, completeness of repair, and the patient's overall condition. The perforation was localized, contamination was minimal, and repair was completed with a two-layer closure by a surgeon; therefore, we considered continuation feasible.

Delayed diagnosis is associated with worse outcomes after bowel injury. In a systematic review of 604 bowel injuries during gynecologic laparoscopy, timing of diagnosis was reported for 375 injuries; among these, diagnosis was delayed by more than one day in 154 (41%), and all five reported deaths occurred in patients with delayed diagnosis [1]. In our patient, recognition of the injury immediately after camera insertion, when bowel mucosa was directly visualized, likely contributed to the favorable outcome. In addition, an injury caused by an initial trocar may be a through-and-through injury; therefore, the procedure should not end after repair of only the lesion first seen. Systematic examination of the bowel to exclude additional injuries is important.

Revised access technique

In response to this event, we revised our initial access technique for subsequent procedures in our institute. Fanning et al. reported that dilating the subcutaneous tissue, fascia, and muscle with a hemostat after the skin incision reduced the median force required for trocar insertion from 6.5 lb to 3.3 lb [11]. In our department, after making the umbilical skin incision, we now clearly identify the fascial defect, dilate it adequately with a hemostat, and elevate the abdominal wall. We also separate peritoneal entry from trocar insertion: the peritoneum is first opened using a method selected according to the preoperative assessment, and the trocar is then introduced through the pre-existing opening. As an additional measure, we changed from an 8-mm blunt obturator to an 8-mm bladeless obturator (optical) (470359, Intuitive Surgical, Inc.) with the intention of reducing the axial force required during trocar insertion. Although this obturator can be used for optical entry, we did not adopt this approach because of concern that use with the robotic endoscope could allow the obturator to advance farther into the abdominal cavity than intended. In our revised protocol, the obturator is inserted only after the peritoneum has been opened separately.

In our revised protocol, the method used to open the peritoneum is selected according to the preoperative assessment. During the admission examination, the periumbilical visceral slide is qualitatively assessed by transabdominal ultrasonography. When sliding is clearly preserved, adhesions are considered less likely but are not regarded as completely excluded; the abdominal wall is therefore adequately elevated, and the peritoneum is opened bluntly with a small instrument such as a mosquito hemostat while limiting the depth of advancement as much as possible. When sliding is absent or equivocal and adhesion of bowel or omentum to the abdominal wall is suspected, the peritoneum is grasped and opened sharply under direct vision in a manner consistent with the Hasson open technique [12]. This policy represents a safety measure introduced in our department after the present event, and no comparative data demonstrate its superiority. Ultrasonographic findings are used only as adjunctive information when selecting the access method, and a negative finding alone is not regarded as a guarantee of safety.

## Conclusions

The absence of previous abdominal surgery does not guarantee safe umbilical access. In our case, although the transverse colon was not directly adherent to the abdominal wall, it was thought to have been tethered by omentum adherent to the abdominal wall, resulting in colonic injury during initial access through the intervening omentum. Accordingly, the safety of initial access cannot be determined from surgical history alone.

The lesson from this case is not that one access technique or instrument is uniformly superior, but rather that the force and depth required to reach the peritoneal cavity should be controlled as much as possible. In our department, we no longer use the trocar itself to bluntly penetrate the peritoneum. Instead, after adequately opening the fascia, we select either controlled penetration with a small instrument or direct-vision open entry according to the ultrasonographic findings, and insert the trocar only through an already opened tract. Ultrasonography is an adjunctive measure, and a negative finding does not completely exclude adhesions. If bowel injury occurs, immediate recognition and systematic exclusion of additional injuries are essential.
